# Generative design as a tool to analyse the uncertain loading regimes of intricate skeletal structures

**DOI:** 10.1098/rsif.2025.0023

**Published:** 2025-05-14

**Authors:** John Michael Racy, Bart Boom, Adam Summers

**Affiliations:** ^1^ Mechanical Engineering, University of Washington, Seattle, WA, USA; ^2^ Aeronautics and Astronautics, University of Washington, Seattle, WA, USA; ^3^ Aquatic and Fishery Sciences, University of Washington, Seattle, WA, USA

**Keywords:** generative design, skeletal structure, Batoidea

## Abstract

The morphology of a skeletal structure is driven in part by the forces it endures over evolutionary time. We demonstrate that generative design, an iterative engineering tool, can yield insight into the loading regimes of complex skeletal elements. With only a simple model of the spatial constraints and potential forces seen by a skeletal element, we generated analogous structures. We used this technique to provide insights into the specialization of the radial elements of batoid pectoral fins, a case that would be challenging to analyse by conventional means. Particular configurations of generative designs resulted in structures with a morphology remarkably similar to that of real radials. We suggest that these cases reveal the loading configurations that the real radials have evolved to withstand.

## 1. Introduction

The forces and constraints under which biological structures function are factors that drive morphological specialization. Determining these parameters can be done experimentally (i.e. with implanted strain gauges or digital image correlation) [1,2] or numerically (i.e. free body analysis or finite-element modelling) [3,4]. We offer a third avenue to gaining an insight into the factors that shape morphology-generative design.

**Figure 1.**
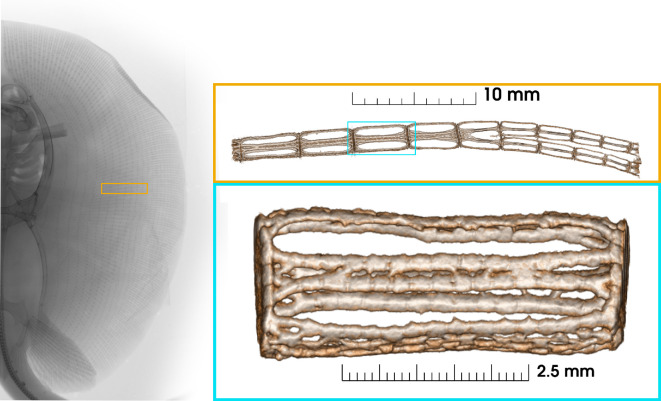
Superior radiograph of a *Potamotrygon* specimen (used with permission of University of Michigan Museum of Zoology. Orange call-out: μCT volume rendering of a section of a fin ray from *Plesiotrygon iwamae* showing the change in structure from proximal to distal and the medial bifurcation. Blue call-out: An individual radial; note the solid end caps and support structure in between.

We are interested in the forces on the skeleton of a stingray as it flaps through the water. The pectoral fin skeleton consists of an array of fin rays that run spanwise from the body to the fin edge (see figure 1). The rays are composed of serially repeating mineralized cartilage elements called radials. The dorsal adductor and ventral abductor muscles that actuate the wing insert at each radial, so there are many layers of muscle and some muscles act across many (15+) joints. There is a chordwise pattern of joints between radials of parallel fin rays which contribute to the locomotor waves required for propulsion. The forces on an individual radial depend not only upon the musculature inserted onto it but also the forces applied to other radials and the torques produced by the actuation of the entire wing. At a finer scale, the mineralization of radials varies with spanwise position. This morphology varies with species and is correlated with the number of waves moving across the wing in steady swimming: mobuliform (*λ* < 0.5) and rajiform (λ > 1) [5,6]. The loading regime on the pectoral fins of stingrays is complex, but we can infer something of the direction, if not the magnitude of forces, on the elements. This is not a problem that can be approached experimentally because the trabecular cartilage of the wing skeleton is not amenable to implantation of strain gauges, especially due to the size of the radials and complexity of surrounding musculature. Finite-element modelling could be used to resolve the precise response of a particular radial to a particular load (Force+Morphology⇒Response). Instead, we applied generative design in search of broader insights into how varied loading regimes, and practical constraints on geometry and material, lead to diverse morphologies (Force+Constraint⇒Morphology). Our goal was explicitly not to precisely analyse a few individual radials, but to improve our understanding of radials in general, and to demonstrate a novel tool for biomechanics.

Generative design for structures is an engineering tool for rapidly producing solutions to loading problems that can minimize local strain, total strain or mass. Structures produced by generative design have rounded forms and large voids reminiscent of trabecular bone, which has earned them the moniker ‘alien bones’ [7]. Generative design starts with a set of geometric constraints, with areas that cannot be filled with material, and areas that must be included in the final design. The user further specifies boundary conditions that allow or restrict movement in particular axes or directions, and a set of forces and moments that can either act at a point or be distributed across elements. These inputs seed an algorithm, currently all proprietary and opaque in their particulars, which explores thousands of possible solutions to converge on those that minimize mass for a given stiffness or maximize stiffness for a given mass. Optimization in traditional design processes relies on the expertise of the designer; in contrast, generative design uses only a repetitive, algorithm-driven approach to produce optimal designs independent of intuition [8]. This iterative approach mimics some aspects of evolutionary algorithms that have been used to find counterintuitive, workable solutions to mechanical problems. For example, [9] work on vertebral stiffness and number using Tadro, a swimming robot.

We set out to compare the morphology of stingray radials and a generative design created within analogous constraints. This case study documents a new way to relate loading regime to morphology that may be broadly applicable to understanding trabecular arrangement in bone, biological trusses and the erosion patterns in the bones of poorly mineralized fishes. Our goals were fourfold: (i) establish the geometric constraints and loading conditions for producing structures that mimic stingray radials using generative design; (ii) compare the results of generative designs produced with different loading and constraints; (iii) assess whether generative designs appear similar to particular radials and (iv) compare the volume fraction of radials and the generative designs to assess this quantifying parameter as a proxy for morphology.

## 2. Material and methods

### 2.1. Anatomical specimens

We imaged material from preserved specimens of two batoid fishes from the collection of the Biomechanics Lab at Friday Harbor Labs. The long-tailed river stingray, *Plesiotrygon iwamae* [10], is an undulatory swimmer with a rounded pectoral fin profile producing multiple wave periods along the wing per flap. The banded eagle ray, *Aetomylaeus nichofii* [11], is an oscillatory swimmer with a triangular fin profile generating a half wave period per flap. We dissected narrow strips of pectoral fin from both specimens so that μCT scans of proximal and distal radials could be captured at resolutions of 14.9 μm, for *P. iwamae* and 13.8 μm, for *A. nichofii* on a Bruker Skyscan 1173. For visualization, we generated volume renderings (Mouse_CT preset settings) using the free, open source software three-dimensional Slicer [12].

### 2.2. Generative design

**Figure 2.**
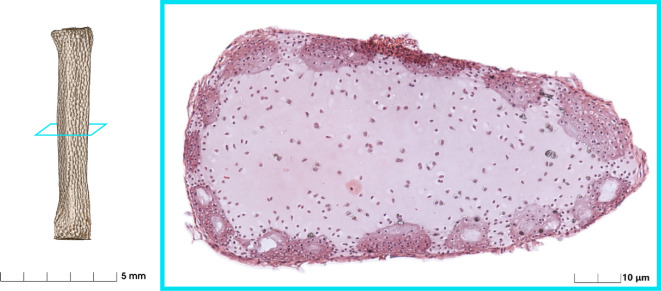
μCT volume rendering of a radial with a histological cross section (dissected from fixed *Aetomylaeus nichofii*, dehydrated in acetone, decalcified with EDTA, paraffin embedded, sliced at 11 μm, stained with haematoxylin and eosin, imaged on Zeiss Axio). Mineralized tesserae are the dense regions around the perimeter. The core of the radial is soft and unmineralized.

**Figure 3.**
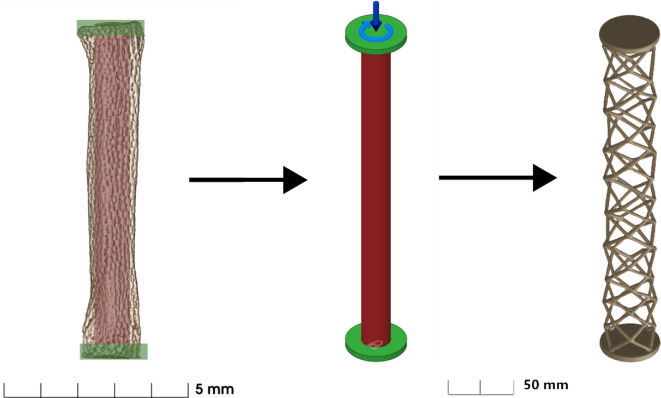
Left: example radial from *Aetomylaeus nichofii* with highlighted regions corresponding to the preserved (green) and obstructed (red) regions of the generative design set-up. Middle: thin disks are preserved. Central cylinder is the obstruction. Lock symbol (bottom) is the constrained end. Arrows (top) are the applied force and moment. Right: example resultant structure.

Fusion 360 (Autodesk, San Francisco) was used under educational license for computer-aided design and solving the generative design cases. The simulation requires geometric dimensions, loadings and material properties. Histology shows that the cartilage of the radials only mineralized on the outer surface (perichondrium) of the radial. Furthermore, all radials have end caps (see figure 2). Therefore, we specified the following geometric constraints to simulate the stingray radial—a pair of circular endcaps with a central cylindrical obstruction (see figure 3).

We constrained one end cap position in all three directions and three rotations, then added various combinations of compressive and torsional loads to the other end. The length between the ends was 300 mm, the ends were each 5 mm thick, the diameter of the ends was 50 mm and the diameter of the cylindrical obstruction was 24 mm. This size and aspect ratio were chosen arbitrarily, as we found the increase in absolute size made generative design resolve more quickly and consistently. The program works at a resolution defined on a unitless low (0) to high (10) scale in the study settings. Models which are too small end up lacking in complexity as the program is unable to generate any smaller geometry. Compressive loads ranged from 100 to 10 000 N, and the torsional loads from 0.1 to 100 Nm. The large loading ranges were chosen to bracket a relevant regime for the loading of the batoid radials, since the actual forces on the radials is unknown.

For all trials, gravity was suppressed and the objective was set to ‘Minimize Mass’ with a safety factor of 1.00 and the displacement option deselected, as in our case we were not defining a maximum allowed deflection under loading. The ‘manufacturing method’ was set as ‘Additive’ only and was oriented in the Z+ axis with 45° overhang and 3 mm allowed minimum thickness. We posit that using an additive procedure to set a minimum thickness simulates the ‘building blocks’ of a structure. Radials are made up of mineralized tesserae meaning that a minimum thickness larger than cell size exists. We used HP three-dimensional CB PA 12 plastic from the Fusion 360 Additive Material Library. This material has an elastic modulus of 1700 MPa which is about 1000 times higher then radials 0.71−4.85 MPa, and it has similar elongation at break of 15–20% [13,14]. We added a slotted cylinder around the central obstacle body defined as a ‘Starting Shape’ to speed up the solving process. With no starting shape, the initial iteration is determined based on the position and geometry of the preserved bodies. A starting shape circumvents that first step. We ran the program under compression and torsion individually to establish the effect of each loading regime on geometry.

We tested a quantitative method for comparing the results of a generative study to a morphology using unitless volume fraction representing space filled.


$$V_{f}=\frac{V_{generated/mineralized}}{V_{total}}$$

For the generative models, the total volume is defined to be that of the entire cylindrical region including the end caps. For the radials, a total volume was approximated as the cylinder found using the averages of the major and minor axes of both end caps.

## 3. Results

Generative design revealed a material efficient solution to resist compressive loads: simple struts at low loads, which branch near the end caps. As compressive load increased the simple struts changed into more complexly divided shapes at the ends. This branching is remarkably similar to those visible near the end caps of the catenated radials of *P. iwamae*, particularly in the simpler distal structure. Under torsion, material was redistributed radially, returning irregularly twisted elements even at low loading. The structures became more regular with increasing load forming an interlocking web of spiraling segments from one end cap to the other. Adding compression and torsion had different effects depending on the proportion of compressive load. At high compressive loads, the basic design was recognizably the strutted compressive design with a slight twist. But, when the compressive loads were lower the addition of even a small torsional load led to a complete surface network of interconnected struts. This network is reminiscent of the arrangement of tessera in the crustal radials of *A. nichofii*.

The volume fraction percentage reflecting the amount of mineralization/stiff material in the total volume of the generative designs revealed more material in structures with higher stress (table 1). The two fishes showed opposite trends to one another, with the oscillator being more mineralized distally and the undulator being the reverse. About a quarter of the radial space was take up by mineral (except in one case). In the pure compression models, there was twice the material when load was increased by an order of magnitude from 100 N; then a fivefold increase for the next order of magnitude increase. The volume fraction percent did not come close to the CT scanned animals until the highest compressive load. Torsion alone required more material than compression, and the combination required large (≈ 50–100%) gains in material.

**Table 1.** Volume fraction percentages for the radials and the generative designs.

radial/generative design		Vf (%)
distal	*P. iwamae*	22.48%
	*A. nichofii*	29.35%
proximal	*P. iwamae*	26.90%
	*A. nichofii*	13.46%
compression (N)	100	3.85%
	1000	6.54%
	10 000	29.31%
torsion (Nm)	0.1	6.68%
	1	5.18%
	10	7.73%
combination (N, Nm)	100, 0.1	5.68%
	100, 1	5.94%
	100, 10	8.15%
	1000, 0.1	6.50%
	1000, 1	6.58%
	1000, 10	9.96%

**Figure 4.**
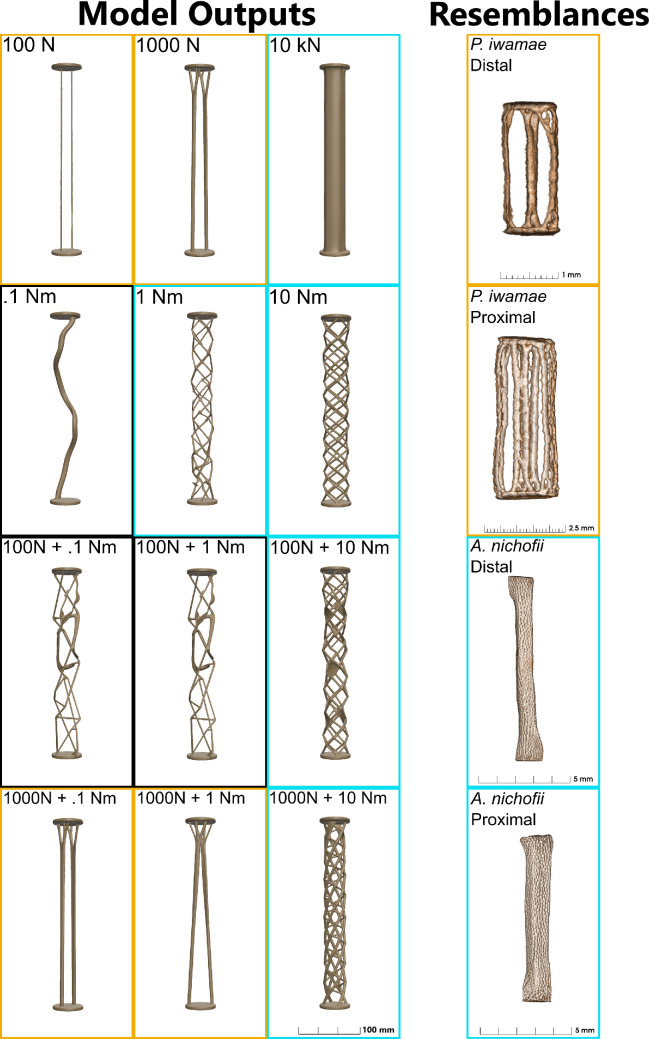
Left panel: structures produced by the generative design algorithm under compression, torsion or combination loading. Borders: orange exhibits traits of catenated morphology, blue has crustal characteristics. Right column: μCT volume renderings of radials.

## 4. Discussion

Generative design knows nothing about stingrays, flapping locomotion or evolution, and yet, a simple set of constraints and hypothetical forces generates complex shapes that are immediately identifiable as similar to living systems (figure 4). We suggest that the generative designs have, in fact, revealed some of the loading regimes of the stingray skeleton. The elements in the tips of the wing, loaded by one or just a few muscles, resist only in compressive load. The anatomy of these distal radials is free-standing columns of mineral, just as we see in the generative models of a simple compressive load. But when a small torsional load is added to the compressive load, the shape of the generative model changes radically to resist the twist displaying interconnectivity between the columns. This new form is recognizable as the mineralized anatomy of a radial near the central axis of a stingray. These medial radials, loaded by the combined shortening of the muscles attached to each of the more distal radials, must be under both compressive and torsional load, although this has never been measured. We believe that this demonstrates that generative design is a useful tool for generating hypotheses about loading in biological structures by generating shapes with known loading regimens and comparing them with the actual morphology of the skeletal element.

Our generative model produces crustal-like structures more readily when its loading regime includes a higher proportion of torsion. Catenated-like structures are favoured when torsion is not significant. We suppose this is because (i) the radials of *P. iwamae* experience greater torsional loading than those of *A. nichofii*; (ii) that the distal and proximal radials of *P. iwamae* both experience torsion-dominated loading and (iii) that the proximal radials of *A. nichofii* experience a torsion-dominated regime, while its distal radials experience a compression-dominant one.

The volume fraction parameter works to determine the amount of material present in a radial or produced by generative design. For pure compression, where material distribution is a simple problem, it does provide a useful proxy for similarity between morphology and generative design. It gives a proxy on the scaling between material properties and size. If the material properties were known, it would be possible to estimate what loads are present *in vivo*. The choice of quantifying parameter will become more difficult when applying this method to other biological systems as the choices for parameters and coordinate systems will depend entirely on the geometry in question. Through further refinement of the material properties, dimensional relations, force-constraint configurations and quantifying parameters, it would be possible to iterate the generative model to find close approximations of the exact forces these radials experience. Currently, finite-element methods may be better suited to such a precise task, but the insight into the relevant loading regimes gained from this analysis using generative design would expedite the set-up of the FEA model.

Caveats to this method’s application are as follows: (i) Because biological materials are extremely locally variable, data on the material properties of a particular structure may be sparse. Acquiring that data and using it to create custom material profiles would vastly improve the quality of findings from a generative design model. (ii) The scales of biological structures, and those favoured by engineering generative design tools, often differ greatly. Appropriate scaling can pose a challenge for resolving solutions and must be determined on a case-by-case basis. (iii) Many iterations are often required to find good solutions, and computation can be slow. And, (iv) this method’s greatest limitation is that it is static-only. Many structures in nature are compliant, contain flexure mechanisms or perform their function under dynamic excitation. Acknowledging the need for continued development to alleviate these caveats, we believe that generative design offers a new tool for biomechanists to analyse the static mechanics, loading regimes and forms of complex skeletal morphology.

## Data Availability

Data are available from Dryad: [15].
